# Supplementary material for: Role and mechanism of NCAPD3 in promoting malignant behaviors in gastric cancer
Source: Front Pharmacol. 2024 Apr 22;15:1341039. doi: 10.3389/fphar.2024.1341039 (PMC11070777; doi:10.3389/fphar.2024.1341039)
Supplement: Supplementary file 11 [file DataSheet2.ZIP › GSEA/GO_CC/my_analysis.Gsea.1599461408869/GO_LAMELLIPODIUM.html]

Details for gene set GO\_LAMELLIPODIUM[GSEA]

|  || Dataset | filtered\_dataset.sample\_info.cls#WT\_versus\_NCAPD3\_MUT |
| Phenotype | sample\_info.cls#WT\_versus\_NCAPD3\_MUT |
| Upregulated in class | WT |
| GeneSet | GO\_LAMELLIPODIUM |
| Enrichment Score (ES) | 0.3364418 |
| Normalized Enrichment Score (NES) | 1.2752342 |
| Nominal p-value | 0.19120136 |
| FDR q-value | 0.68420047 |
| FWER p-Value | 1.0 |
Table: GSEA Results Summary

  

Fig 1: Enrichment plot: GO\_LAMELLIPODIUM      
 Profile of the Running ES Score & Positions of GeneSet Members on the Rank Ordered List

  

| SYMBOL | TITLE | RANK IN GENE LIST | RANK METRIC SCORE | RUNNING ES | CORE ENRICHMENT || 1 | 323 | APBB2 | 35 | 0.977 | 0.0893 | Yes |
| 2 | 23380 | SRGAP2 | 142 | 0.757 | 0.1019 | Yes |
| 3 | 7248 | TSC1 | 158 | 0.718 | 0.1752 | Yes |
| 4 | 54434 | SSH1 | 183 | 0.692 | 0.2390 | Yes |
| 5 | 324 | APC | 281 | 0.607 | 0.2406 | Yes |
| 6 | 2241 | FER | 304 | 0.588 | 0.2937 | Yes |
| 7 | 960 | CD44 | 339 | 0.573 | 0.3364 | Yes |
| 8 | 4771 | NF2 | 428 | 0.516 | 0.3338 | No |
| 9 | 57679 | ALS2 | 656 | 0.399 | 0.2178 | No |
| 10 | 5311 | PKD2 | 708 | 0.372 | 0.2248 | No |
| 11 | 1605 | DAG1 | 823 | 0.304 | 0.1788 | No |
| 12 | 7429 | VIL1 | 827 | 0.299 | 0.2116 | No |
| 13 | 130271 | PLEKHH2 | 1012 | -0.412 | 0.1281 | No |
| 14 | 4651 | MYO10 | 1213 | -0.588 | 0.0537 | No |
| 15 | 4739 | NEDD9 | 1322 | -0.741 | 0.0630 | No |
Table: GSEA details [plain text format]

  

Fig 2: GO\_LAMELLIPODIUM      
 Blue-Pink O' Gram in the Space of the Analyzed GeneSet

  

Fig 3: GO\_LAMELLIPODIUM: Random ES distribution      
 Gene set null distribution of ES for **GO\_LAMELLIPODIUM**
